# Deregulation of Polycomb Repressive Complex-2 in Mantle Cell Lymphoma Confers Growth Advantage by Epigenetic Suppression of *cdkn2b*

**DOI:** 10.3389/fonc.2020.01226

**Published:** 2020-07-24

**Authors:** Christos Demosthenous, Shiv K. Gupta, Jing Sun, Yongsen Wang, Tammy P. Troska, Mamta Gupta

**Affiliations:** ^1^Division of Hematology, Rochester, MN, United States; ^2^Department of Radiation Oncology, Mayo Clinic, Rochester, MN, United States; ^3^Department of Biochemistry and Molecular Medicine, School of Medicine and Health Sciences, GW Cancer Center, Washington, DC, United States

**Keywords:** MCL, PRC2, EZH2, H3K27me3, cdkn2b

## Abstract

The polycomb repressive complex 2 (PRC2) maintains the transcriptional repression of target genes through its catalytic component enhancer of zeste homolog 2 (EZH2). Through modulating critical gene expression, EZH2 also plays a role in cancer development and progression by promoting cancer cell survival and invasion. Mutations in EZH2 are prevalent in certain B-cell lymphoma subtypes such as diffuse large cell lymphoma and follicular lymphoma; while no EZH2 mutation has been reported in the mantle cell lymphoma (MCL). Here we demonstrate that the PRC2 components EZH2, EED and SUZ12 are upregulated in the MCL cells as compared to normal B-cells. Moreover, stably transfected cells with wild-type EZH2 or-EED showed increased cell growth and H3K27-trimehtylation. However, unlike wild-type EZH2, ectopic expression of a deletion construct of EZH2 (EZH2^Δ550−738^ lacking SET domain) had no growth advantage over control cells. Pharmacological inhibition of EZH2 suppressed H3K27me3 and had significant inhibitory effect on cell growth and colony forming capacity (*p* < 0.05) of MCL cells, and this effect was more or less comparable to the anti-proliferative effects of EZH2 inhibition in cells harboring EZH2-mutation. Mechanistically, EZH2 appears to downregulate expression of *cdkn2b* gene via enhanced H3K27me3, a well-known suppressive epigenetic mark, at the *cdkn2b* promoter region. Overall, these results highlight that deregulation of PRC2/EZH2 is associated with epigenetic suppression of *cdkn2b* in MCL, and in part responsible for increased cell growth, thus the EZH2 inhibitors may have therapeutic potential in the patients with MCL.

## Introduction

Mantle cell lymphoma (MCL) is an aggressive form of B cell lymphoma, which accounts for ~6% of all non-Hodgkin lymphomas (NHL) (1, 2). Treatment outcome in MCL appears to be improving with help of novel targeted agents including ibrutinib (3, 4). Nevertheless, patients do relapse, often with emergence of drug resistance, which remains a critical barrier to the current treatment strategies. Therefore, identifying alternative approaches to overcome drug resistance in the relapsed MCL is an urgent unmet medical need. Polycomb repressive complex 2 (PRC2), is an important epigenetic regulator that comprises three main components: enhancer of zeste homolog 2 (EZH2), zinc finger-containing SUZ12 and WD40-repeat protein EED. EZH2 is the catalytic component of the PRC2 and represents a key a histone methyltransferase primarily involved in transfer of methyl groups to the lysine 27 residue of histone H3 (H3K27) through its C-terminal SET domain (5). Importantly, EZH2 gains histone methyltransferase activity only when it complexes with other 2 subunits, EED and SUZ12 to form PRC2 (6–9).

Overexpression of EZH2 in tumor cells has been implicated in tumor growth, and has inverse correlation with treatment outcome in various cancer types (10–14). In B cell lymphoma, aberrancy of EZH2 has been associated with EZH2 tyrosine 641 (Y641N) mutation seen in ~22% of diffuse large B-cell lymphoma (DLBCL) and 7% of follicular lymphoma (FL) patients (15–17). However, EZH2 mutations have not been reported in the MCL patient samples or human MCL cell lines (18, 19). Apart from pathogenic mutations, genetic lesions of EZH2, including copy number amplification, chromosomal gain or loss have been reported in DLBCL and FL (20, 21). Besides genetic alterations, the epigenetic regulation of EZH2 expression and its activity via promoter methylation, microRNA and lncRNA have also been reported (22). Notably, we have previously shown that lncRNA ROR1-AS1 expressed in MCL, can modulate activity of EZH2 (23). Given the importance of EZH2 dysregulation in cancers including lymphoma, there is growing interest in EZH2 inhibitors and a number of EZH2 inhibitors are under various stages of clinical development (24). Although several studies have previously shown oncogenic role of mutant-EZH2 found in DLBCL and FL (15, 16, 25), studies aimed at understanding the role of wild type (wt) EZH2 in other lymphoma subtypes such as MCL, are lacking. The present study was designed to comprehensively evaluate role of PRC2 in MCL and to study the implications of targeting H3K27 methylation events through pharmacological inhibitors of EZH2.

## Materials and Methods

### Cell Lines

Human MCL lines Jeko, Mino, Granta, JVM2, and Z138 purchased from ATCC (Manassas, VA) were grown in RPMI supplemented with 10% Fetal Bovine Serum (FBS) and 1% streptomycin/penicillin. HEK293T cell line was obtained from Open Biosystems (Huntsville, AL, USA) and was cultured in the DMEM supplemented with 10% Fetal Bovine Serum (FBS). Cell lines were authenticated by STR Profiling performed at the ATCC and Genetica (Burlington, NC USA). CD19+ B-cells were sorted form the peripheral blood mononuclear cells isolated from healthy donors using EasySep isolation kit (STEMCELL Technologies).

### Antibodies and Drugs

Antibodies against EZH2 (cat#4905), SUZ12 (cat#3737), histone H3 (cat#4499) and H3K27me3 (cat#9733) were purchased from the Cell Signaling Technologies (Beverly, MA, USA). Antoibodies to H3K27me1 (cat#ab175037), H3K27me2 (cat#ab24684) and EED (cat#ab231812) were from Abcam (Cambridge, MA, USA), while the anti-β-Actin (cat#sc47778) antibody was procured from Santa Cruz Biotechnology (Dallas, TX, USA). EZH2 specific inhibitors GSK343, GSK126 and methyltransferase inhibitor DZNep were purchased from the Sigma-Aldrich (St Louis, MO, USA).

### Plasmid Construction and Stable Transfection

Full length cDNA encoding EZH2^WT^ was PCR amplified using custom made forward and reverse primers. The PCR fragments were cloned into an intermediate vector pCR2.1-TOPO-TA (Thermo-Fisher), sequence verified and subsequently cloned into pLEX-MCS lentiviral vector. The viral particles were packaged using Viral System Protocols (Dharmacon) and HEK293 cells were virally transduced and selected with 500 ng/ml puromycin as previously described (26).

### Transient Cell Transfection

HEK293-T cells were transfected with 5.0 μg of plasmids (PCDNA3.1 empty vector or carrying EZH2^WT^ or EZH2^Δ550−738^) using lipofectamine-2000 (Invitrogen, Grand Island, NY, USA) for 24 h as previously described (26).

### Co-immunoprecipitation (Co-IP) and Western Blotting

For the co-immunoprecipitation assay, 5 μg of EZH2 antibody was added to the lysates and incubated with continuous shaking at 4°C, the immune-precipitates were then pulled down using protein G-Agarose beads, washed four times with the lysis buffer and denatured. The protein acrylamide gel electrophoresis and western blotting was performed as previously described (27). Briefly, the proteins resolved by SDS-PAGE were transferred to PVDF membrane. The membranes were incubated in blocking solution 5% non-fat milk dissolved in PBS, 0.1% Tween-20 (PBST), and subsequently with the indicated primary antibodies at 4°C overnight. The membranes were then washed in PBST and incubated with HRP-conjugated secondary antibodies for 1 h at room temperature and the signal developed by enhanced chemiluminescent reagent and captured by the autoradiography. Densitometry on original western blots was quantified by ImageJ software and the signal was normalized to corresponding control lane.

### Quantitative Reverse Transcription-PCR (Q-PCR)

Total RNA was extracted using TRIzol reagent (Invitrogen, Carlsbad, CA) and Q-PCR was performed as previously described (28). Relative mRNA expression was calculated using the ΔΔCt method (29) after normalization, the primers used were as follows (all sequences from 5 to 3′):

β-actin -forward primer: GCCGCCAGCTCACCAT

β-actin -reverse primer: TCGATGGGGTACTTCAGGGT

*cdkn2b*-forward primer: GGACTAGTGGAGAAGGTGCG

*cdkn2b*-reverse primer: GGGC GCTGCCCATCATCATG

*cdkn2a*-forward primer: GAAGGTCCCTCAGACATCCCC

*cdkn2a*-reverse primer: CCCTGTAGGACCTTCGGTGAC

### Chromatin Immunoprecipitation (ChIP) Assay

ChIP assays were carried out using Simple ChIP® Enzymatic Chromatin IP Kit (9003, Cell Signaling Technology) using anti-H3K27me3 antibody and control IgG as per instruction manual. Briefly, the nuclei isolated from Mino or JVM2 cell lines cultured in absence or presence of 5 μM GSK126, were processed as per instruction manual. The immuno-precipitated DNA and the input samples were analyzed by quantitative PCR using the following primers:

*cdkn2b*-forward primer: 5′- CCTTCCCTGTCCAGGTGGATTT-3′,

*cdkn2b*-reverse primer: 5′-ACCTTAGCTCTGACTCCTCATCC-3 ′.

### Proliferation and Survival Assays

For thymidine incorporation assay, MCL cells were cultured for 48 h at 37°C in 96-well plates and the thymidine incorporation or MTT assays were performed as previously described (27, 30). For assessment of cell survival, 5 × 10^5^ cells per well were cultured in the absence or presence of various drug doses for 48 h and subsequently analyzed by flow cytometry using Annexin V and propidium iodide as previously described (27).

### Colony-Forming Unit (CFU) Assay

For CFU assay, 2 × 10^3^ cells suspended in 50 μl culture medium were added to 0.5 ml Methocult^TM^ H4230 (STEMCELL, Vancouver, Canada) with or without EZH2 inhibitor per well in 24-well plates and incubated at 37°C for 10 days.

### Statistics

The data presented is the mean ± standard error from 3 independent experiments. An unpaired Student's *T*-test was used for statistical comparisons and *p* < 0.05 considered significant.

## Results

### Upregulation of PRC2 Components in MCL Cells

**Figure 1:**
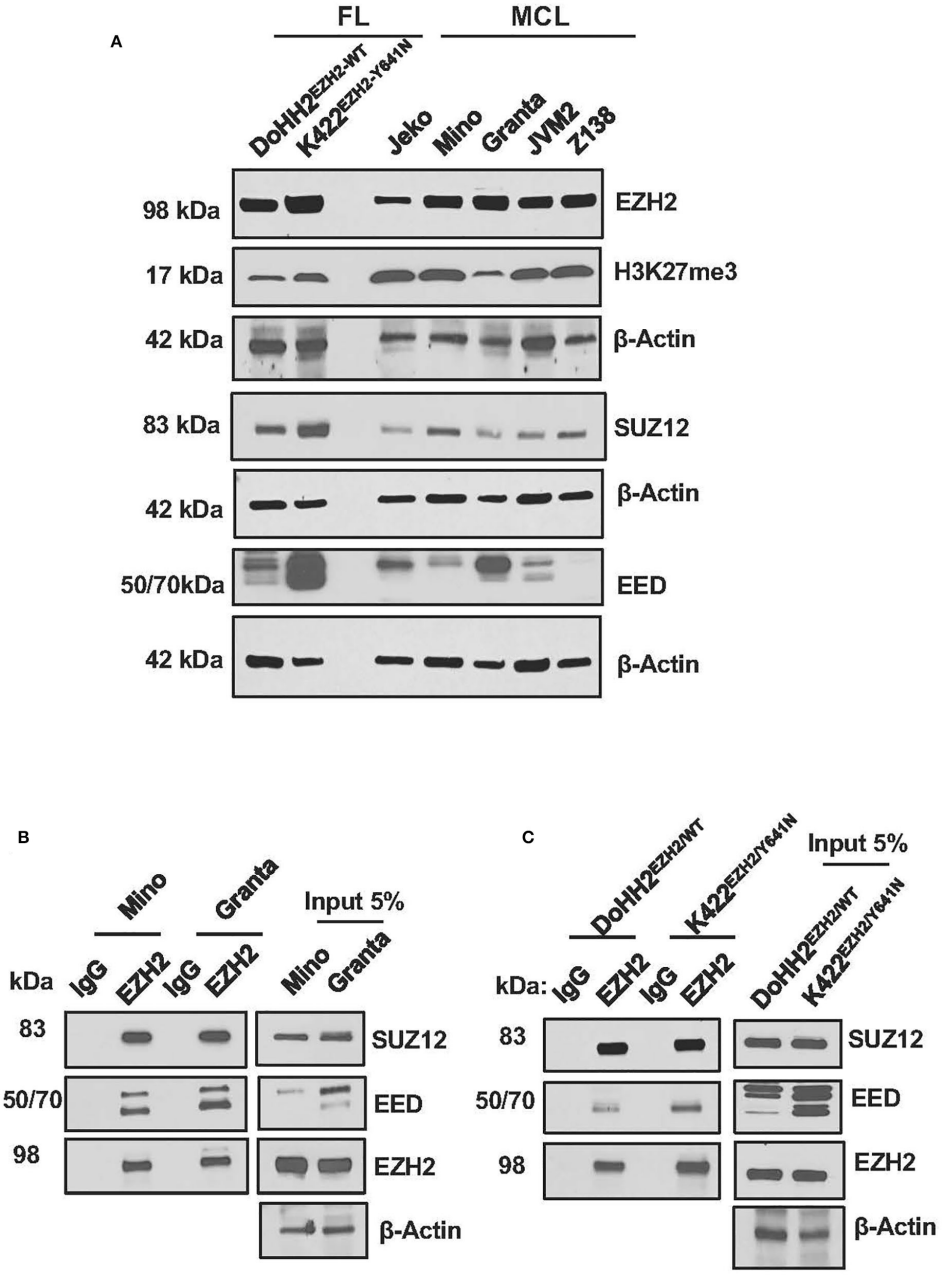
Expression of the PRC2 components in MCL and FL cells. **(A)** Western blot images showing expression of PRC2 components EZH2, SUZ12, and EED in lysates of MCL and FL cell lines, β-Actin was used as a loading control; levels of H3K27-trimethylation (H3K27me3) were detected to assess PRC2 activity, where Histone H3 served as a loading control. **(B,C)** immunoblots showing enrichment of SUZ12 and EED in proteins immuno-precipitated with anti-EZH2 antibody or control IgG from lysates of MCL cell lines, Mino and Granta, carrying WT EZH2 **(B)**, or FL cell lines, DOHH2 and Karpas-422, carrying WT and mutant EZH2, respectively **(C)**.

PRC2 is an epigenetic suppressive complex and plays an important role in modulating gene transcription throughout the stages of development and in various diseases including lymphoma (16, 31–33). To understand potential role of PRC2 in MCL, we first analyzed expression of PRC2 components in MCL cell lines and comparing with FL cell lines, Karpas 422 and DOHH2 differing by EZH2-mutation status. The MCL cell lines used here have been previously described to express a wild-type EZH2 (EZH2^WT^), while FL cell lines, Karpas 422 and DOHH2 are known to express EZH2^mutant^ and EZH2^WT^, respectively (18, 34). Consistent with dysregulation of PRC2 in several human cancers, a robust expression of EZH2, SUZ12, and EED proteins was seen on the western blots for all MCL cell lines analyzed (Figure 1A). However, FL cell line Karpas 422, carrying EZH2^Y641N^ mutation exhibited slightly higher levels of EZH2, H3K27me3, SUZ12, and EED than the FL cell line DOHH2 carrying EZH2^WT^, and had PRC2 components comparable to that of MCL cell lines (Figure 1A). Interestingly, the expression of PRC2 components EZH2, EED, and SUZ12 was lower in CD19+ normal B-cells as compared to that in lymphoma cell lines. Moreover, the global level of H3K27-trimethylation in the lymphoma cell lines was more or less similar to that of normal B cells (Supplementary Figure S1). These results indicate that the levels of EZH2, SUZ12, and EED (PRC2 components) are upregulated in lymphoma cells as compared to normal B cells, but this is not the case for global levels of H3K27 tri-methylation.

Confirming the interaction among various components of PRC2, SUZ12, and EED co-immuno-precipitated in an EZH2 pulled down assay using anti-EZH2 antibody, and the pull-down efficiency of PRC2 components with anti-EZH2 antibody was comparable in the MCL and FL cell lines (Figures 1B,C). Overall, these results demonstrate that dysregulation of PRC2 in MCL cells is essentially similar to that in Karpas-422, a FL cell line carrying Y641N mutation in EZH2.

### Effect of Ectopic Expression of EZH2 or EED on Cell Growth and Histone Methylation

**Figure 2:**
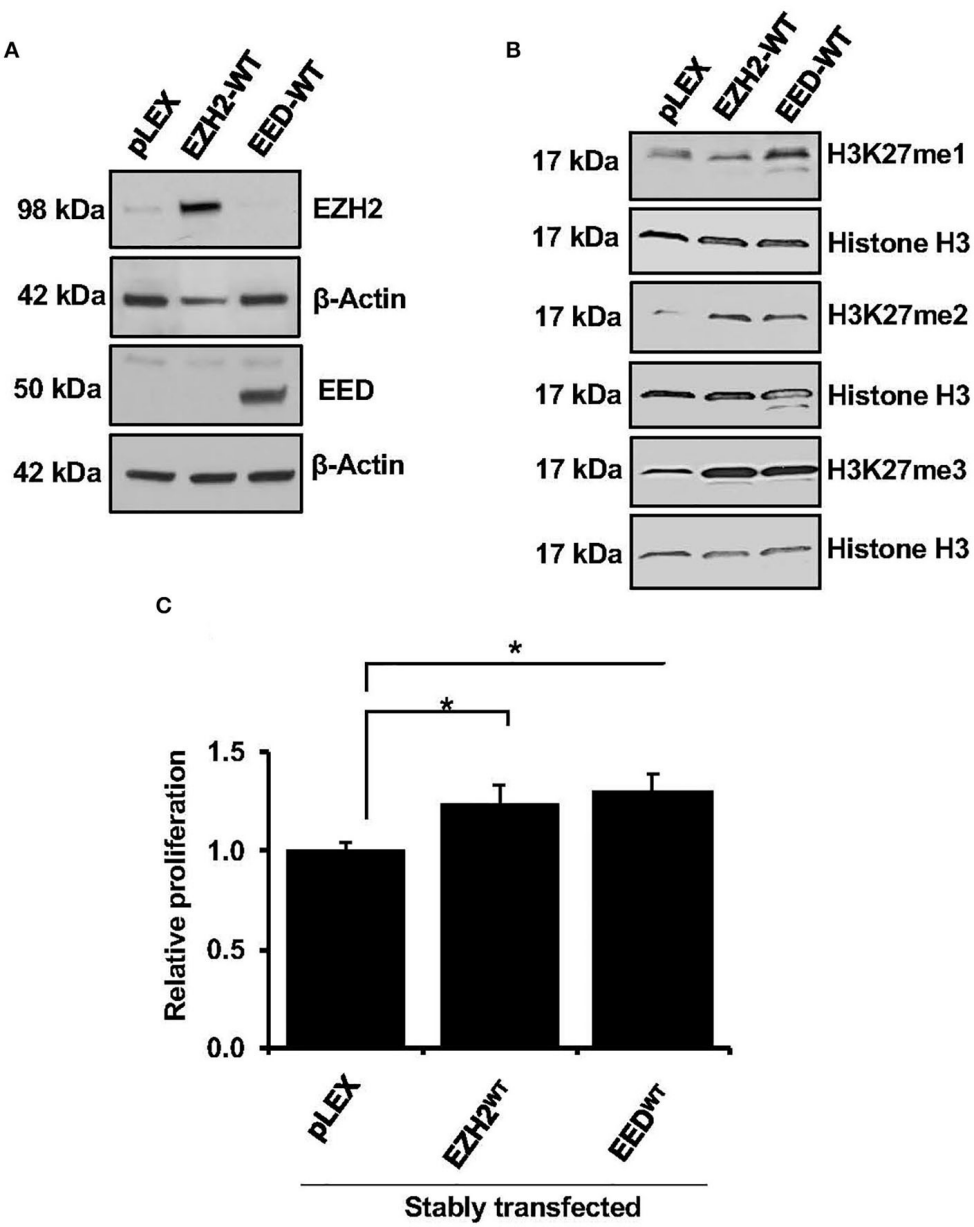
Effect of EZH2 or EED transfection on H3K27-methylation and cell growth. **(A)** Western blot analysis shows overexpression of EZH2 and EED in stably transfected HEK293T cells, β-Actin was used as a loading control. **(B)** western blot images showing levels of H3K27me1, H3K27me2, and H3K27me3 in HEK293T cells transfected with an empty vector (pLEX) or the vector containing full length EZH2 or EED, histone H3 was used as loading control. **(C)** Bar graphs showing relative cell proliferation as determined by MTT assay in the HEK293T cells transfected with empty vector (pLEX) or vector containing EZH2 or EED, bars represent mean ± SD from 3 different experiments (*p < 0.05).

EZH2 is the enzymatic subunit of PRC2 complex (5), whereas EED, is an essential component required for allosteric activation of methyltransferase activity (35). To elucidate effects of EZH2 and EED overexpression on methyltransferase activity and cell growth, full length EZH2 and EED cDNA were expressed in the HEK-293T cells. Since we were unable to achieve stable overexpression of EZH2 or EED in the MCL cell lines using lentiviral system despite multiple attempts, EZH2 or EED expression vectors were transduced in HEK-293 cells (Figure 2A). Overexpression of full length EZH2 or EED (EZH2^WT^ or EED^WT^) led to a markedly increased H3K27me3 and to a lesser extent the di-methylation of H3K27 (H3K27me2) marks as compared to corresponding empty vector controls. However, increased level of H3K27me1 was only observed with overexpression of EED but not with EZH2 overexpression (Figure 2B). These results indicate that overexpression of EZH2 or EED is associated with dysregulation of histone methyltransferase activity of PRC2. Next, we assessed the effect of the EZH2 or EED overexpression on cell growth. Results from an MTT assay showed that overexpression of EED or EZH2 significantly (*p* = 0.05) promotes cell proliferation as compared an empty vector (Figure 2C). Overall, these results suggest that dysregulation of methyltransferase activity of PRC2 by overexpression of EZH2 or EED can promote cell growth.

### Pro-growth Function of PRC2 Depends on the EZH2 SET Domain

**Figure 3:**
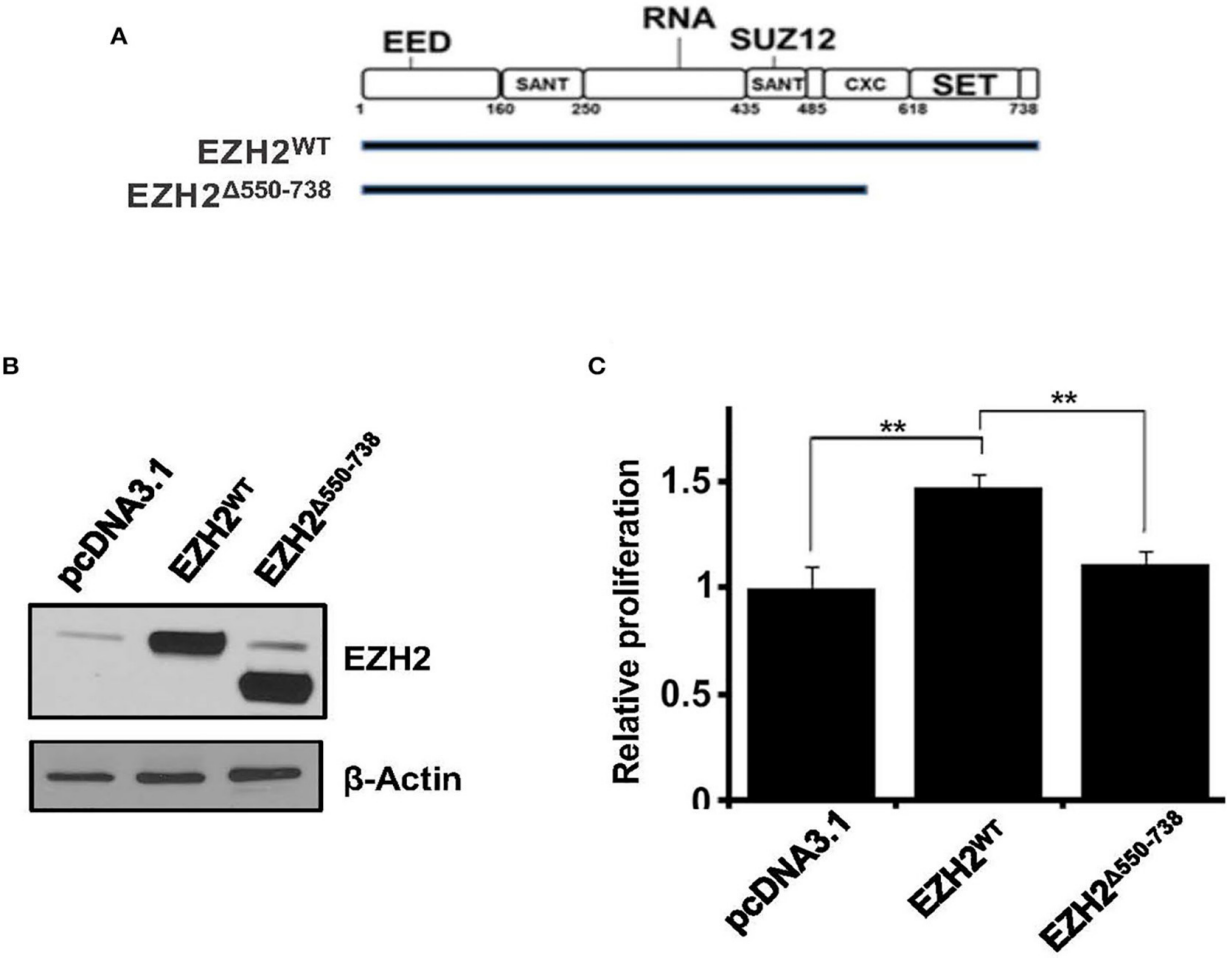
Effect of ectopic expression of truncated EZH2 or EED on histone methylation and cell growth. **(A)** Schematic depiction of full-length and truncated EZH2 constructs. **(B)** Representative western blot images showing overexpression of full length (EZH2^WT^) or truncated EZH2 (EZH2^Δ550−738^). **(C)** graphs showing relative cell proliferation (as determined by the MTT assay) in the HEK293T cells transfected with an empty vector (pcDNA3.1) or pcDNA3.1 carrying full length EZH2 (EZH2^WT^) or truncated EZH2 (EZH2^Δ550−738^). Bars represent mean ± SD from 3 different experiments (**p < 0.01).

EZH2 is the catalytic subunit of the PRC2, and its C-terminal SET domain exhibits methyltransferase activity. To elucidate the biological significance of EZH2 SET-domain in cell proliferation we cloned full-length EZH2 (EZH2^WT^) or a SET-domain deletion construct (EZH2^Δ550−738^) into pCDNA3.1 mammalian expression vector and transiently transfected into the HEK293 cells (Figures 3A,B). We then sought to determine the effect of ectopically expressed EZH2^WT^ and EZH2^Δ550−738^ constructs on cell growth by MTT assay. Unlike, full-length EZH2, which led to significant increase in cell growth, overexpression of EZH2^Δ550−738^, had no significant growth advantage over cells transfected with an empty vector (Figure 3C). Overall these results suggest that EZH2, overexpression has the potential to increase cell growth.

### Growth Inhibitory Effects of Pharmacological Inhibition of EZH2 in MCL Cells

**Figure 4:**
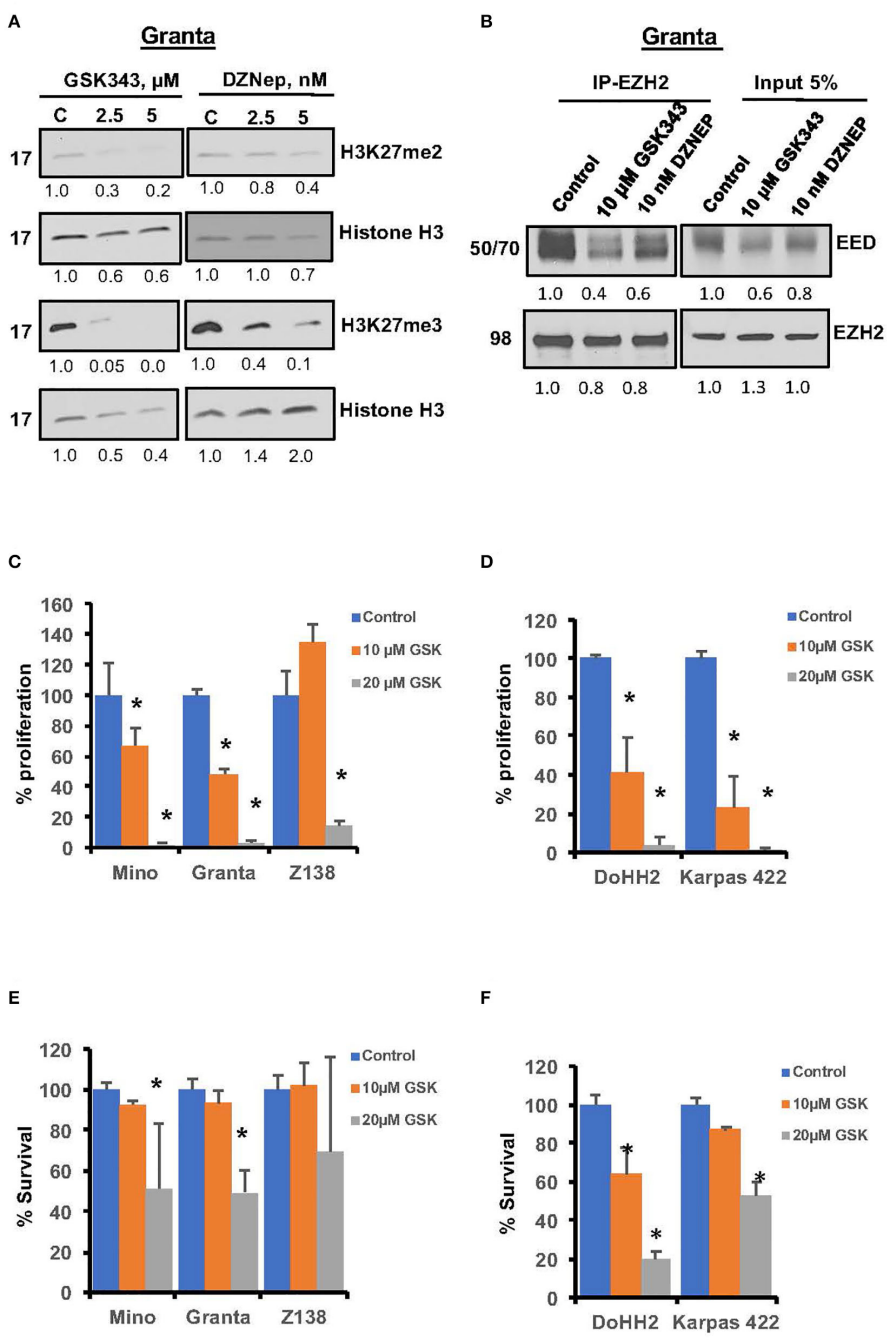
Effects of pharmacological inhibition of EZH2 on growth and PRC2 integrity in lymphoma cells. **(A)** Representative western blot images showing H3K27me2 and H3K27me3 in Granta cells treated with GSK343 or DZNep used at indicated concentrations for 48 h, β-Actin and the histone H3 were used as loading controls. The numbers underlining each western blot image represent relative band intensity as compared to the control lane in same blot. **(B)** Representative images showing levels of EED or EZH2 in proteins immuno-precipitates using either IgG or anti-EZH2 antibody along with 5% input from the lysates of Granta cells treated with EZH2 inhibitors as indicated. **(C–F)** MCL cell lines (left panel) and FL cells (right panel) were treated with various concentrations of EZH2 inhibitor GSK343 for 72 h. Proliferation **(C,D)** and survival **(E,F)** was assessed by thymidine incorporation and Annexin V/PI exclusion, respectively. Bars represent mean ± SD from 3 different experiments (*P < 0.05).

There is growing interest in clinical development of EZH2 inhibitors in lymphoma, and selective EZH2 inhibitor have shown promising activity in EZH2 mutant cells (34, 36). The effect of EZH2 inhibition on suppressive histone marks H3K27me2/3 was assessed in Granta cells. Western blot analysis of H3K27me2/3 showed marked reduction in H3K27me3 and H3K27me2 marks after GSK343 treatment; however, the effect of GSK343 was relatively more pronounced on H3K27me3 than H3K27me2 epigenetic marks (Figure 4A). Similarly, DZNep, a global methyltransferase inhibitor decreased H3K27me3 but had no appreciable effect on H3K27me2 histone marks. Furthermore, EZH2 inhibition by the GSK343 or DZNep decreased PRC2 formation by prohibiting interaction between EZH2 and EED as determined by co-immunoprecipitation assay (Figure 4B). Overall these results suggest that PRC2 can be targeted in MCL with GSK343, which is relatively more effective than DZNep.

We then sought to analyze effects of EZH2 specific inhibitors GSK343 or GSK126, on proliferation and survival of MCL (Mino, Granta and Z138) and FL cell lines (DOHH2 carrying EZH2^WT^ and Karpas 422 carrying mutant EZH2^Y641N^). Treatment with 10 μM GSK343 reduced proliferation of Mino and Granta cells by 25 and 40%, respectively, while 20 μM GSK343 completely obliterated growth of these cell lines (Figure 4C). Similar anti-proliferative effects of GSK126, a different EZH2 inhibitor, were seen with Mino and JVM2 cell lines (data not shown). Interestingly, although GSK343 rendered more prominent anti-proliferative effects in FL cells than in MCL cells, there was no significant difference in anti-proliferative activity of GSK343 among FL cell lines, DOHH2^EZH2WT^ vs. Karpas 422^EZH2/Y641N^ (Figure 4D) indicating an inherent hypersensitivity to EZH2 inhibitors in FL cells that is unrelated to EZH2 mutation. In a cell survival assay, 10 μM GSK343 had no significant effect, while 20 μM GSK343 decreased survival by ~45–50% in Mino and Granta cell lines, but no significant inhibitory effect was observed in Z138 cell line (Figure 4E). Consistent with a more pronounced anti-proliferative effects EZH2 inhibition in FL cells, treatment with GSK343 also had more pronounced effect on cell survival in FL cell lines, where 20 μM GSK343 reduced survival to only 20 and 40% in DOHH2 and Karpas422 cell lines, respectively (Figure 4F). To further confirm growth inhibitory effect of EZH2 blockade in MCL cells, we performed colony-forming unit (CFU) assay. Consistent with results from proliferation and survival assays, CFU assay showed significant reduction in colony formation in the presence of GSK126 as compared to untreated controls (Supplementary Figures S2A,B). Overall, these results suggest that PRC2 dysregulation regardless of pathogenic EZH2 mutations, can enhance growth potential in MCL and FL cell lines with or without EZH2 mutations. These results suggest that deregulation of PRC2 and its oncogenic potential is much more prevalent in lymphoma subtypes than previously realized.

### Epigenetic Effect of EZH2 Inhibition on Cell Cycle Regulatory Genes in MCL

**Figure 5:**
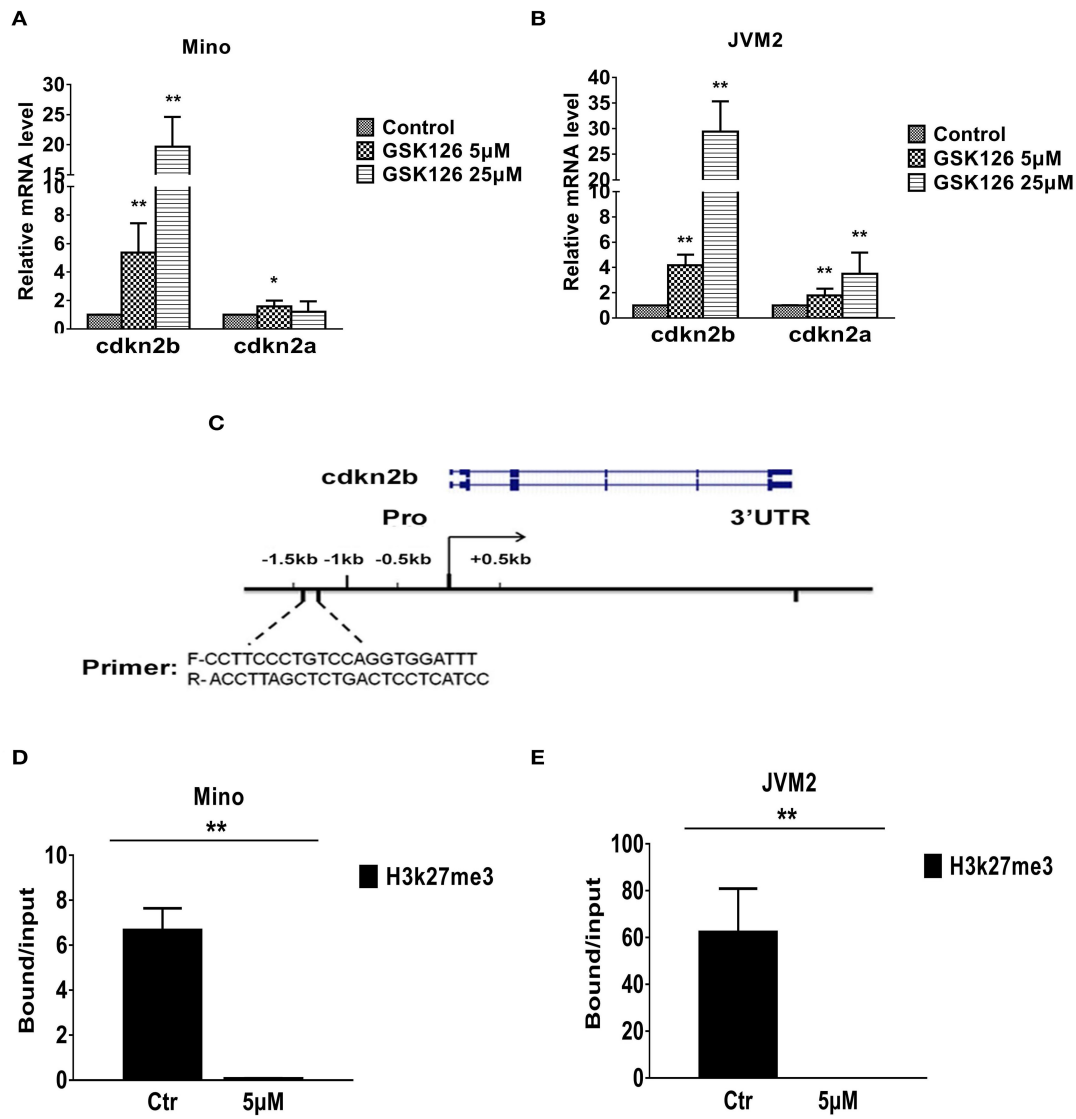
Effect of EZH2 inhibition on expression of tumor suppressor genes in MCL cell lines. **(A,B)** Bar graphs showing relative mRNA transcript of *cdkn2a* and *cdkn2b* as determined by QRT-PCR in Mino **(A)** or JVM2 **(B)** MCL cell lines treated with EZH2 inhibitor for 48 h. **(C)**. Schematic diagram of promoter region of c*dkn2b* used in ChIP assays, sequence of primers and location of targeted region has been depicted. **(D,E)** Bar graphs showing fold enrichment of promoter region of c*dkn2b* gene after ChIP with anti-H3K27me3 antibody as compared to input in Mino **(D)** or JVM2 **(E)** cells cultured in absence or presence of GSK126. Bars represent mean ± SD from 3 different experiments, *p < 0.05, and **p < 0.01 for the comparison of *cdkn2b* amplicon in ChIP with H3K27me3 antibody in control versus GSK126 treated cells.

To understand how EZH2 confers growth advantage in the MCL cell lines, we focused on our attention to the cell cycle regulators, which are most commonly disrupted or deregulated either by homozygous deletion or epigenetic silencing via promoter hyper-methylation of *cdkn2b* (p15), and/or *cdkn2a (*p16) loci (37, 38). To understand potential role of EZH2 in regulation of *cdkn2a* and/or *cdkn2b*, we first determined the impact of EZH2 inhibition on expression of *cdkn2a* and *cdkn2b* in Mino and JVM2 cells. Interestingly, EZH2 inhibition have modest (2–4-folds) but statistically significant upregulation of *cdkn2a*, while the effect on expression of the *cdkn2b* was highly remarkably upregulated e (~20–30-folds, *p* = 0.005 as compared to control) (Figures 5A,B). These analyses indicate that EZH2 (PRC2) can promote growth of lymphoma cells via suppression of *cdkn2b* and/or *cdkn2a*.

To understand the mechanism of EZH2 mediated regulation of *cdkn2b*, we performed chromatin immunoprecipitation (ChIP) assays on MCL cells cultured in absence or presence of GSK126 using the anti-H3K27me3 antibody or IgG control, and the primers against *cdkn2b* promoter as depicted in Figure 5C. Results from ChIP assay showed that the inhibition of EZH2 by GSK126 significantly (*p* = 0.005) decreased H3K27me3 marks in the promoter region of *cdkn2b* both in Mino and JVM2 cell lines (Figures 5D,E). Overall, these results suggest that EZH2 promotes H3K27me3 repressive histone marks on promoter region of *cdkn2b*, which in turn can repress *cdkn2b* gene.

## Discussion

PRC2 is an epigenetic suppressive complex, which plays an important role in gene transcription in various diseases (16, 31–33). Histone methyltransferase EZH2 is the catalytic subunit of PRC2, and the tyrosine 641 (Y641) of EZH2 is the most frequently mutated residue, up to 22% of germinal center-derived lymphomas and 24% of FL acquiring mutations at this site (16, 17). These mutations appear to modify enzymatic activity of EZH2 (32, 33). However, such EZH2 activating mutations, have not been reported or identified in the whole exome sequencing data from MCL patient samples (18, 19), as a result, the role of PRC2 and wt-EZH2 in MCL has been historically considered to be limited. However, our results from this study suggest that PRC2 may have tremendous therapeutic implication considering MCL is an aggressive B-cell lymphoma with frequent relapses.

In the current study, we have demonstrated that PRC2 components, EZH2, SUZ12, and EED, are highly overexpressed in MCL cell lines as compared to the normal B-cells isolated from the peripheral blood of healthy donors. These findings are consistent with the previous study by others (39, 40). To take the concept of PRC2 overexpression one step further, immunoprecipitation studies showed that histone methyltransferase complex is enzymatically active in proliferating MCL cells in absence of known EZH2 mutations. Collectively, these data show that PRC2 is aberrantly active in MCL cells. Consistent with prior studies suggesting pro-tumorigenic role of PRC2 (34, 35), overexpression of wt-EZH2 was associated with enhanced proliferation. Patients with MCL requiring treatment, receive either combination of intensive chemotherapy backbone incorporating high dose cytarabine and rituximab or a combination of bendamustine and rituximab (41). Despite this standard of care treatment, patients do relapse and often with emergence of drug resistance. EZH2 is an attractive drug target in cancer, which has inspired clinical development of EZH2 inhibitors to target mutant EZH2 (42, 43). Our results suggest that EZH2 inhibitors can inhibit H3K27 tri-methylation by disrupting interaction between EZH2 and EED in MCL cell harboring wt-EZH2. Importantly, treatment with EZH2 inhibitors led to significant growth inhibition and cell killing, which was comparable to the activity of EZH2 inhibitors in lymphoma cells harboring gain-of-function mutations in EZH2. Considering the deregulation of PRC2 is associated with growth potential of lymphoma cells, targeting PRC2 with EZH2 inhibitors is a reasonable therapeutic approach to aggressive lymphomas. However, considering important role of PRC2 in B-cell development, use of EZH2 inhibitors will need caution to prevent potential adverse effects on hematopoietic homeostasis. Nevertheless, our results are in accordance with a prior study performed by Zhang et al. (44) who demonstrated reduced survival of MCL cells using DZNep (44).

Studies in MCL patient samples have shown DNA hyper-methylation of key genes implicated in cell cycle regulation, such as *cdkn2a* and *cdkn2b* (37, 38, 45). Our study showed that EZH2 inhibition can restore *cdkn2b* expression by altering epigenetic landscape of histone methylation marks, and support the notion that *cdkn2b* is a direct target of PRC2/EZH2 in MCL. Our results are in line with prior study where *cdkn2b* repression was associated with epigenetic alterations in the form of DNA methylation as well as EZH2 mediated suppressive histone marks that were reversed by the epigenetic drugs (46). Consistent with the later scenario, the abundance of H3K27me3 suppressive histone marks were detected in promoter region of *cdkn2b* in MCL cells, which was reduced by the EZH2 inhibition. Whether EZH2 can act independently or requires DNA hyper-methylation to silence *cdkn2b* in MCL is not clear. Nevertheless, the data reported here indicate that overexpression of EZH2 (with or without activating mutations) may confer a similar PRC2 addiction previously reported for the DLBCL or FL cells with mutant-EZH2. These observations suggest that there can be broader scope for translation of EZH2 inhibitors in MCL and other subtypes of non-Hodgkin-lymphoma. These findings also identify *cdkn2b* as an epigenetic target of EZH2, and provide the rationale for launching clinical trials of EZH2 inhibitors in relapsed or treatment refractory mantle cell lymphoma patients or other B-cell malignancies.

## Data Availability Statement

The raw data supporting the conclusions of this article will be made available by the authors, without undue reservation.

## Author Contributions

CD performed most of the research, analyzed the data, made the figures, and wrote the manuscript. SG performed the research and wrote the manuscript. JS, YS, and TT performed the research. MG conceived and designed the study, supervised all aspects of research project, interpreted data, wrote the manuscript, and finalized the figures. All authors contributed to the article and approved the submitted version.

## Conflict of Interest

The authors declare that the research was conducted in the absence of any commercial or financial relationships that could be construed as a potential conflict of interest.
