# Supplementary figures and images for: Deregulation of Polycomb Repressive Complex-2 in Mantle Cell Lymphoma Confers Growth Advantage by Epigenetic Suppression of cdkn2b
Source: Front Oncol. 2020 Jul 24;10:1226. doi: 10.3389/fonc.2020.01226 (PMC7396700; doi:10.3389/fonc.2020.01226)

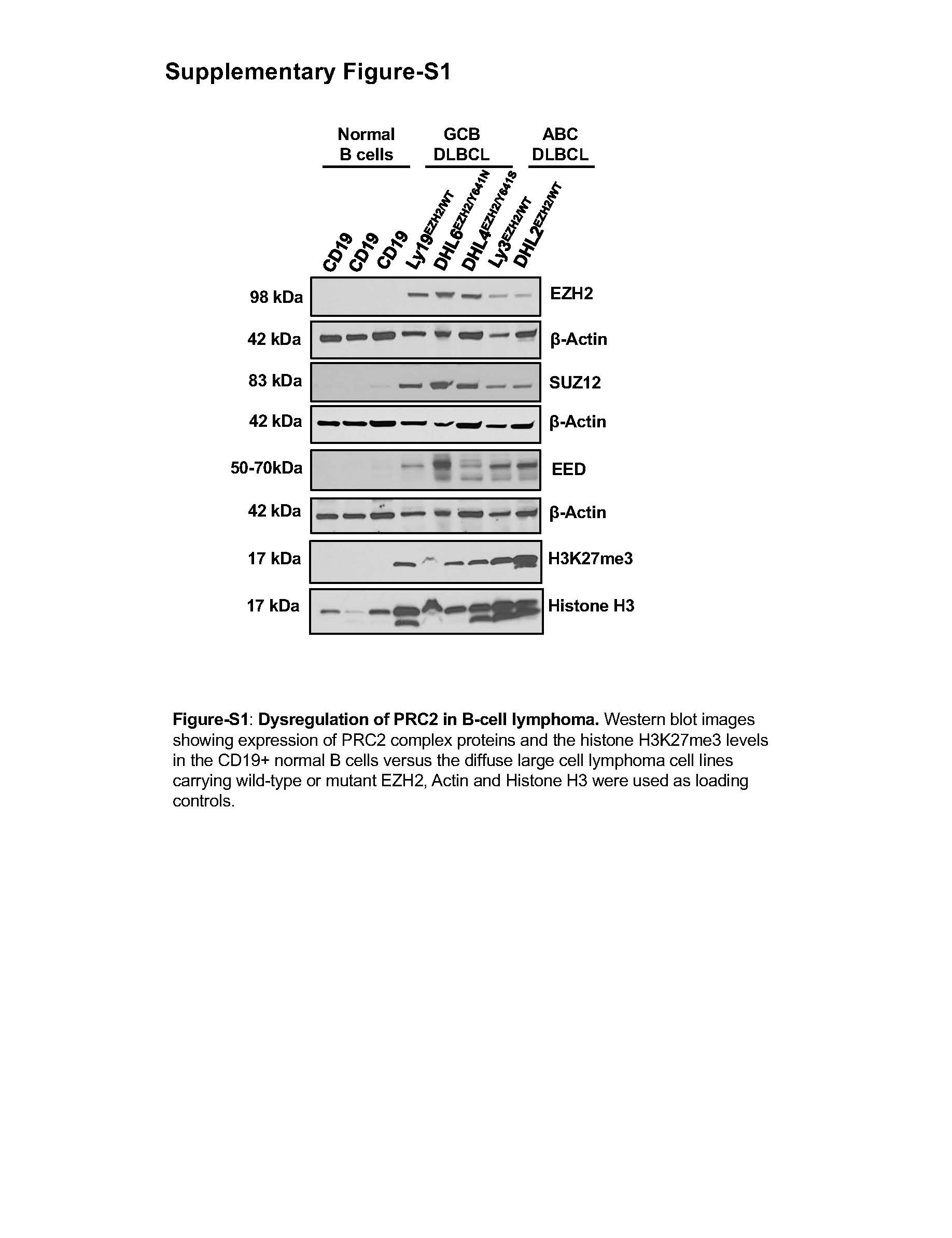

Supplement: Supplementary file 1 [file Image_1.JPEG]

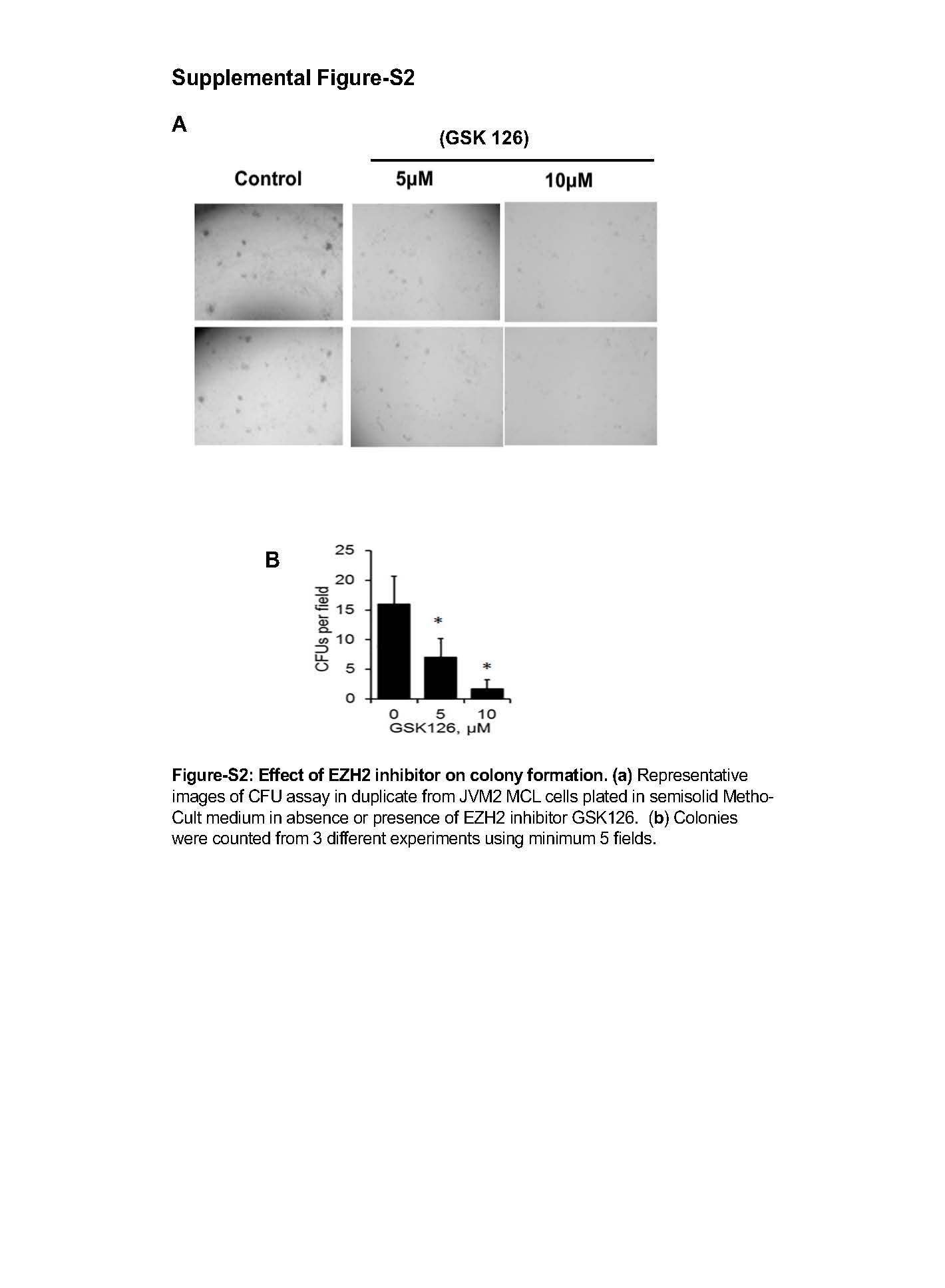

Supplement: Supplementary file 2 [file Image_2.JPEG]
